# Plant polyprenols reduce demyelination and recover impaired oligodendrogenesis and neurogenesis in the cuprizone murine model of multiple sclerosis

**DOI:** 10.1002/ptr.6327

**Published:** 2019-03-12

**Authors:** Marina Y. Khodanovich, Anna O. Pishchelko, Valentina Y. Glazacheva, Edgar S. Pan, Elena P. Krutenkova, Vladimir B. Trusov, Vasily L. Yarnykh

**Affiliations:** ^1^ Laboratory of Neurobiology Tomsk State University Tomsk Russian Federation; ^2^ Prenolica Limited (formerly Solagran Limited), Biotechnology Company Melbourne Victoria Australia; ^3^ Department of Radiology University of Washington Seattle WA USA

**Keywords:** cuprizone model, demyelination, long‐chain isoprenoid alcohols, neurogenesis, oligodendrogenesis, plant polyprenols

## Abstract

Recent studies showed hepatoprotective, neuroprotective, and immunomodulatory properties of polyprenols isolated from the green verdure of Picea abies *(L.) Karst*. This study aimed to investigate effects of polyprenols on oligodendrogenesis, neurogenesis, and myelin content in the cuprizone demyelination model. Demyelination was induced by 0.5% cuprizone in CD‐1 mice during 10 weeks. Nine cuprizone‐treated animals received daily injections of polyprenols intraperitoneally at a dose of 12‐mg/kg body weight during Weeks 6–10. Nine control animals and other nine cuprizone‐treated received sham oil injections. At Week 10, brain sections were stained for myelin basic protein, neuro‐glial antigen‐2, and doublecortin to evaluate demyelination, oligodendrogenesis, and neurogenesis. Cuprizone administration caused a decrease in myelin basic protein in the corpus callosum, cortex, hippocampus, and the caudate putamen compared with the controls. Oligodendrogenesis was increased, and neurogenesis in the subventricular zone and the dentate gyrus of the hippocampus was decreased in the cuprizone‐treated group compared with the controls. Mice treated with cuprizone and polyprenols did not show significant demyelination and differences in oligodendrogenesis and neurogenesis as compared with the controls. Our results suggest that polyprenols can halt demyelination, restore impaired neurogenesis, and mitigate reactive overproduction of oligodendrocytes caused by cuprizone neurotoxicity.

## 1 INTRODUCTION

Polyprenols are bioactive long‐chain isoprenoid alcohols that occur in various plants. Unlike more popular in the area of nutrition polyphenols, which contain multiple phenolic structural units, polyprenols consist of hydrophilic and hydrophobic parts: a hydroxyl group and a long unsaturated, mainly of poly‐cis configuration, isoprenyl chain. Depending on the source, chain length of natural polyprenols varies from 6 to 40 isoprene units (Roslinska, Walinska, Swiezewska, & Chojnacki, 2002; Zhang et al., 2015).

A small amount of polyprenols in the form of free alcohols, carboxylic esters, and phosphate esters is present in cell membranes (Zhang et al., 2015). Bacterial and some plant membranes contain unsaturated polyisoprenols (polyprenols), whereas unicellular eukaryotes, fungi, animal, and some plant tissues contain saturated polyisoprenols (dolichols; Roslinska et al., 2002). After administration in small animals, polyprenols were found in high concentrations in the liver, kidneys, and lungs and in lesser concentration in the brain, spleen, and other organs (Chojnacki & Dallner, 1988). Upon subfractionation of the liver, most of the polyisoprenoids were recovered in the mitochondrial‐lysosomal fraction (Jakobsson, Swiezewska, Chojnacki, & Dallner, 1989). In the liver, polyprenols are mostly modified to metabolically more active forms via α‐saturation and phosphorylation by a specific kinase (Chojnacki & Dallner, 1988). The dolichol phosphate cycle plays an important role in biosynthesis of membrane and intracellular glycoproteins. Phosphates of polyisoprenoids, due to their hydrophobic properties, act as carriers of glycosyl residues across membranes during glycosylation reactions (Walinska, 2004), C‐ and O‐protein mannosylation, and cell wall biosynthesis (Hartley & Imperiali, 2012). Due to a large number of unsaturated carbon bonds, polyprenols are prone to oxidation, being precursors of a variety of compounds, including terpenes and steroids (Walinska, 2004).

Polyprenols and their metabolites have attracted considerable attention due to their proven hepatoprotective, antioxidant, neuroprotective, immunomodulating, and proliferative activity. Yang, Wang, Ye, and Li (2011) and J. Yu et al. (2012) demonstrated hepatoprotective effect of polyprenols in the model of tetrachloride‐induced hepatic damage using liver function tests and histology. The authors associated the effect with a reduction of oxidative damage, downregulation of pro‐fibrogenic stimuli, inhibition of activation of hepatic stellate cells, and protection of hepatocytes. In a pilot clinical study, 30‐day polyprenol treatment of patients with chronic alcoholism (Soultanov et al., 2010) resulted in significant improvement of blood biochemistry according to the clinical liver, pancreas, and kidney tests.

The effect of polyprenols of natural origin was investigated in animal models of Alzheimer's disease. In particular, Fedotova et al. (2016) and Fedotova, Soultanov, Nikitina, Roschin, and Ordayn (2012) showed that 4‐week administration of polyprenols ameliorates cognitive impairment caused by intracerebroventricular injection of β‐amyloid (Acs et al., 2009; Bakker & Ludwin, 1987; Barnett & Prineas, 2004; Deshmukh et al., 2013; Festing, 2014; Iwasa et al., 2014; Khodanovich et al., 2016; Lucchinetti et al., 2000; Vega‐Riquer, Mendez‐Victoriano, Morales‐Luckie, & Gonzalez‐Perez, 2017; Veto et al., 2010; Q. Yu et al., 2017) in the passive avoidance paradigm, Morris water maze, and an open‐field test. Soultanov et al. (2017) using the same animal model demonstrated positive effect of polyprenol administration on the depression‐like behavior in the forced swimming test. Moreover, these authors (Fedotova et al., 2016; Soultanov et al., 2017) also showed synergic positive effect of testosterone and polyprenols on β‐amyloid‐induced impairment in gonadectomized rats. A similar effect of polyprenol treatment was described by Wang, He, Yan, Zheng, and Liu (2014) on the d‐galactose mouse model of Alzheimer's disease. In this study, polyprenols improved the d‐galactose‐induced cognitive impairment in Morris water maze, passive and active avoidance tests, and an open‐field test via enhancing oxidative defense and affecting generation and dissimilation of Aβ‐related enzymes. In a clinical electroencephalography study (Soultanov et al., 2010), polyprenol treatment of patients with chronic alcoholism enhanced the alpha‐rhythm power and decreased pathological excitation in the frontal brain areas.

Mild immunomodulating and antiviral properties of polyprenyl phosphates were shown by Pronin et al. (2002). The authors reported inhibition of an early phase of interleukin 1 and Con A interaction in spleen cells, lypoxigenase activity, and expression of interleukin 2 receptors by polyprenyl phosphates. At the same time, phosprenyl stimulated natural killer cell activity and early TNF‐α production (Pronin et al., 2002).

Due to participation in glycan biosynthesis and N‐linked protein glycosylation, polyprenyl phosphates and dolichyl phosphate are important regulators of cell proliferation. It was shown that the rate of dolichyl phosphate and glycoprotein synthesis is linked to the growth rate of Chinese hamster ovary cells and cell division (Hartley & Imperiali, 2012; Swiezewska & Danikiewicz, 2005). Moreover, the studies have confirmed that dolichyl phosphate is a rate‐limiting substrate in N‐linked glycosylation and is thereby a key factor in cellular development (Hartley & Imperiali, 2012; Swiezewska & Danikiewicz, 2005). Inhibition of polyisoprenol biosynthesis resulted in abnormal gastrulation, which correlated with the inability of the cell to produce glycoproteins. Addition of exogenous dolichol allowed for normal gastrulation, suggesting that dolichyl phosphate is a limiting reagent for N‐linked glycosylation of proteins and subsequent cellular transformations (Hartley & Imperiali, 2012).

We hypothesize that the spectrum of biological activity of polyprenols may be of significant interest in treatment of multiple sclerosis (MS), a chronic inflammatory demyelinating and neurodegenerative disorder. In addition to traditional anti‐inflammatory MS therapies, the use of complementary and alternative herbal medicine gained substantial attention over the past years as a way to alleviate neurological deficit and augment neuroprotection (Mojaverrostami, Bojnordi, Ghasemi‐Kasman, Ebrahimzadeh, & Hamidabadi, 2018; Zarshenas, Ansari, Dadbakhsh, & Mohammadi, 2018). The search for new agents that could enable the restoration of damaged myelin and prevent neurodegeneration is of crucial importance for further progress in the treatment of this condition. Given the effect of polyprenols in various neurological and cognitive conditions demonstrated in animal models and in humans and the fact that toxicity of pure polyprenols is virtually absent (Wang, Yuan, Li, Zhang, & Ye, 2015), these substances can be of interest for preclinical testing in animal models of MS.

The objective of this study was to investigate the treatment with polyprenols in an established animal model of MS, cuprizone‐induced demyelination in mice. Cuprizone [oxalic acid bis(cyclohexylidene hydrazide)] is a copper chelator that causes reversible widespread demyelination in the murine brain structures characterized by primary oligodendrocyte depletion, microglial activation, and astrogliosis (Kipp, Clarner, Dang, Copray, & Beyer, 2009; Praet, Guglielmetti, Berneman, Van der Linden, & Ponsaerts, 2014) resembling type III human MS lesions (Lucchinetti et al., 2000) that are believed to represent the earliest stage of MS pathology (Barnett & Prineas, 2004). Certain features of this model, particularly after prolonged cuprizone exposure, such as prominent demyelination of gray matter (Khodanovich et al., 2017) and neuroaxonal degeneration (Kipp et al., 2009; Praet et al., 2014), also mimic clinically relevant pathological aspects of normal‐appearing brain tissue damage in chronic and progressive MS. The cuprizone model is commonly considered beneficial for studying mechanisms of demyelination and remyelination in isolation from autoimmune factors and preclinical tests of new interventions for promoting remyelination and neural tissue recovery in MS (Kipp et al., 2009; Praet et al., 2014). In line with previous studies utilizing the cuprizone model to assess the efficacy of therapeutic agents preventing demyelination and promoting remyelination (Acs et al., 2009; Deshmukh et al., 2013; Iwasa et al., 2014; Veto et al., 2010; Zhang et al., 2008), we sought to evaluate a potential effect of polyprenols on the extent of demyelination and restorative processes including neurogenesis and oligodendrogenesis in the cuprizone‐treated mice.

## 2 MATERIALS AND METHODS

### 2.1 Animals

Adult 8‐week‐old male CD‐1 mice (*n* = 27) were obtained from the vivarium of the Institute of Pharmacology of the Siberian Branch of the Russian Academy of Sciences. Although the typically used strain for creating the cuprizone model is C57BL/6 mice (Kipp et al., 2009; Praet et al., 2014), we have chosen the most common in preclinical drug safety and efficacy testing CD‐1 strain (Festing, 2014). Cuprizone‐induced demyelination can be successfully generated in CD‐1 mice, which was demonstrated earlier (Bakker & Ludwin, 1987; Vega‐Riquer et al., 2017; Q. Yu et al., 2017), though lower sensitivity of this strain to cuprizone intoxication was documented (Q. Yu et al., 2017). Only male animals were used because females are known to be less susceptible to cuprizone demyelination (Kipp et al., 2009). The age of the animals at the beginning of the experiment was chosen according to the standard guidelines for the cuprizone model (Kipp et al., 2009; Praet et al., 2014). Animals were housed with a 12‐hr dark–light cycle at a temperature of 21 ± 2°C and humidity of 40 ± 2%. Food and water were provided *ad libitum*. The animals were housed and treated in accordance with the rules adopted by the European Convention for the Protection of Vertebrate Animals used for Experimental and Other Scientific Purposes. All animal procedures were approved by the Ethics Committee of the Biological Institute of Tomsk State University.

### 2.2 Substances

Demyelination was induced in the mice with cuprizone (bis(cyclohexanone)oxaldihydrazone, Sigma‐Aldrich, USA). Pharmaceutical‐grade polyprenols (at least 95% polyprenols or long‐chain isoprenoid alcohols, including eight to 18 isoprene residues) were isolated from the green verdure of Picea abies *(L.) Karst* as previously described (Fedotova et al., 2012) and supplied as the registered pharmaceutical form (Ropren®, Prenolica Limited, Melbourne, Australia). The oil vehicle used in this study was 100% refined olive oil (F.lli Ruata S.p.A., Italy).

### 2.3 Experimental design

The experiment was designed to test the hypothesis that polyprenol treatment can halt acute demyelination caused by cuprizone and/or prevent the development of chronic demyelination. After 10 days of quarantine, the animals were randomly divided into three groups, nine mice in each group: control + vehicle, cuprizone + vehicle, and cuprizone + polyprenols. The cuprizone animal model of MS was induced as previously described (Khodanovich et al., 2016; Koutsoudaki et al., 2009; Pott et al., 2009). The cuprizone + vehicle and cuprizone + polyprenols groups were fed with the standard chow diet containing 0.5% cuprizone for 10 weeks. Of note, the cuprizone dosage was at the high end of the dose range typically used in cuprizone studies (0.2–0.5%; Gudi, Gingele, Skripuletz, & Stange, 2014; Kipp et al., 2009; Koutsoudaki et al., 2009; Praet et al., 2014; Q. Yu et al., 2017) in view of its less pronounced toxic effect in CD‐1 mice as compared with C57BL/6 mice (Q. Yu et al., 2017), for which the optimal dose of 0.2% has been suggested (Gudi et al., 2014). A similar dose (0.5–0.6%) was used earlier for the same strain (Bakker & Ludwin, 1987), where it produced visible myelin degradation by the third week and almost complete demyelination after 7–9 weeks of cuprizone administration. The control + vehicle group was fed the regular vivarium chow. The animals in the cuprizone + polyprenols group were given daily intraperitoneal injections of polyprenols at a dose of 12 mg/kg (dissolved in 0.1 ml of oil vehicle) from the sixth week of cuprizone treatment. The animals of the control + vehicle and cuprizone + vehicle groups were injected with the same volume of the vehicle from the sixth week. The timeframe of the experiment was chosen to begin treatment with polyprenols at the peak of acute demyelination, which is achieved from the fifth to sixth week of cuprizone administration according to the literature (Gudi et al., 2014; Kipp et al., 2009; Praet et al., 2014). Continued cuprizone administration typically results in chronic demyelination with nearly complete myelin loss and a substantially reduced remyelination capacity (Gudi et al., 2014; Kipp et al., 2009; Praet et al., 2014). Two days before the end of the experiment, the mice were tested in an open field. After 10 weeks of cuprizone feeding, the mice were transcardially perfused with 4% paraformaldehyde under ether anesthesia. The brains were removed and fixed overnight in paraformaldehyde at 4°C. The brains were cryoprotected in graded concentrations of sucrose in phosphate buffer (24 hr at 10% and 24 hr at 20%) at 4°C and were then frozen in liquid nitrogen and stored at −80°C for further immunofluorescence study.

### 2.4 Open‐field testing

Two days prior to euthanasia, all mice were tested in an open field, consisting of a 50 × 50‐cm^2^ chamber lined by 25 (5 × 5) squares surrounded by 40‐cm high walls. The following parameters of the animal behavior were counted during 5 min of testing: (a) locomotor activity as the number of squared crossed, (b) vertical activity as the number of rearings, (c) grooming activity as the number of complete grooming acts, and (d) the total number of defecations and urinations, which was considered as an indicator of anxiety‐like behavior.

### 2.5 Immunofluorescence

Coronal brain sections of 10‐μm thickness (−1.58 and +0.74 mm from bregma according to the mouse brain atlas; Paxinos & Franklin, 2001) from seven of nine animals of each group were prepared using an HM525 cryostat (Thermo Fisher Scientific, Walldorf, Germany). Sections were immunofluorescence stained for myelin basic protein (MBP, a marker of myelin), neuro‐glial antigen‐2 (NG2, a marker of oligodendrocyte precursor cell [OPC]), and doublecortin (DCX, a marker of immature neurons). The primary antibodies were goat polyclonal anti‐MBP (sc‐13914, Santa Cruz Biotechnology, USA), rabbit polyclonal anti‐NG2 (H‐300; sc‐20162, Santa Cruz Biotechnology), and goat polyclonal anti‐DCX (C‐18; sc‐8066, Santa Cruz Biotechnology). The secondary antibody was donkey anti‐goat AlexaFluor® 488 (green color, code 705‐545‐147, Jackson ImmunoResearch, USA) or donkey anti‐rabbit AlexaFluor® 488 (green color, code 711‐545‐152, Jackson ImmunoResearch).

Slides were covered with mounting medium with DAPI (4′,6‐diamidino‐2‐phenylindole, blue color, nuclear counter stain). From each animal, sections of both the left and right hemispheres were obtained. Photography was performed using an Axio Imager Z2 microscope (Carl Zeiss, Germany) and AxioVision 4.8 (Carl Zeiss) software with a MozaiX module, which enables the creation of whole‐brain images by means of stitching together smaller images. Identical imaging parameters were set for all photographed sections. Additionally, NG2‐stained sections were photographed using a laser confocal microscope LSM 780 NLO (Carl Zeiss).

### 2.6 Image processing

Image analysis was performed using ImageJ software. Regions of interest (ROIs) of standard size were placed on MBP images manually within the central and distal parts of the corpus callosum (100 × 200 μm^2^), motor cortex (200 × 200 μm^2^), center of the caudate putamen (200 × 200 μm^2^), and the hippocampus (200 × 200 μm^2^). Myelin density on the MBP images was quantified in the above structures by measuring the mean intensity in corresponding ROIs. The mean intensities from each structure were averaged across ROIs and across photographs for each animal. Additionally, MBP was quantified from stained sections using the same ROIs and the Otsu thresholding method in the ImageJ implementation. Percentage of the total area of detected objects was used as a surrogate measure of MBP density (Ercan et al., 2017).

Oligodendrogenesis was evaluated in a series of white and gray matter structures including the corpus callosum, cortex, and caudate putamen. ROIs of standard size (100 × 200 μm^2^ for the corpus callosum, 200 × 200 μm^2^ for the cortex and caudate putamen) were placed on NG2‐stained images manually within investigated brain structure maps using a mouse brain atlas. The number of NG2‐positive cells was counted inside each ROI and calculated as a corresponding number of NG2‐positive cells divided by area of ROI. The number of DCX‐positive cells was counted visually in well‐known zones of active neurogenesis of the adult brain—the subgranular zone of the dentate gyrus and the subventricular zone (SVZ). Five brain sections for each animal and each antibody were analyzed. Immuhistological procedures and image analysis were carried out by the researchers blinded to the animal group.

### 2.7 Statistical analysis

All statistical analyses were carried out in Statistica 10.0 for Windows (StatSoft Inc., Tulsa, OK, USA). Mean values and standard errors of all investigated parameters were calculated for each anatomical structure. Normality of the data within animal groups was assessed using the Shapiro–Wilk test. Levene's test was used to assess homogeneity of variances between the groups. No significant deviations from the normal distribution and differences in sample variances were found, and therefore, parametric analyses were used. Behavioral data were compared between the groups using a one‐way repeated‐measures analysis of variance (ANOVA) model. Immunofluorescence data were compared between the control + vehicle, cuprizone + vehicle, and cuprizone + polyprenols groups using a two‐way repeated‐measures ANOVA model (two factors: “group” factor with three levels, repeated‐measures “structure” factor with a number of levels depending on the type of labeling). Post hoc pairwise tests with Tukey's correction for multiple comparisons were performed for each type of ANOVA. Statistical significance was defined as a *p* value less than 0.05.

## 3 RESULTS

### 3.1 Polyprenols decrease cuprizone‐induced behavioral deficits

**Figure 1:**
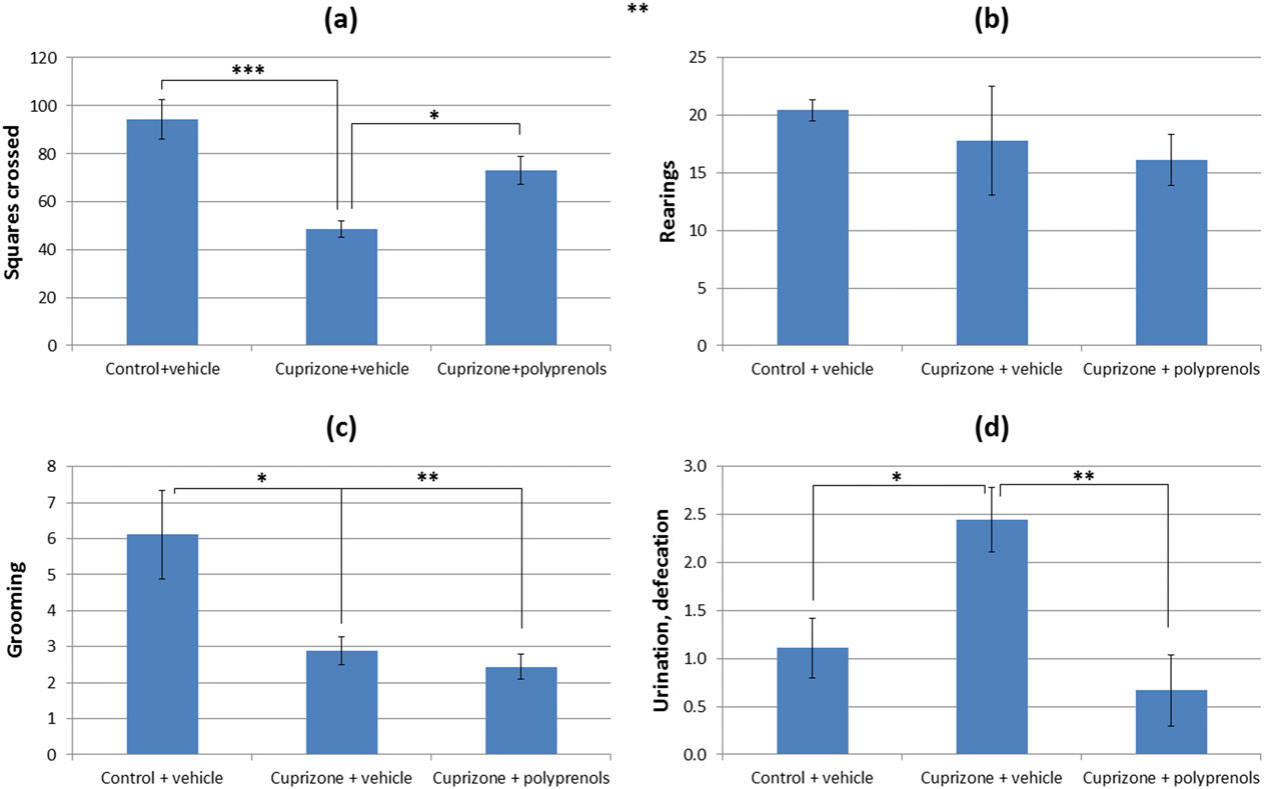
The effect of polyprenols on behavior in an open‐field test in cuprizone‐treated mice. (a) The number of squares crossed. (b) The number of rearings. (c) Grooming activity. (d) The number of defecations and urinations. The significant differences between the groups according to analysis of variance after Tukey's correction for multiple comparisons: ***p < 0.001, **p < 0.01, *p < 0.05. Bars in the panel (b) represent standard errors of mean [Colour figure can be viewed at wileyonlinelibrary.com]

The results of open‐field tests are shown in Figure 1. The animals from the cuprizone + vehicle group had a significantly lower locomotor and grooming activity and a higher level of anxiety‐like behavior compared with controls. Polyprenol treatment significantly increased locomotor activity and completely reversed anxiety‐like behavior caused by cuprizone feeding.

### 3.2 Polyprenols decrease cuprizone‐induced demyelination

**Figure 2:**
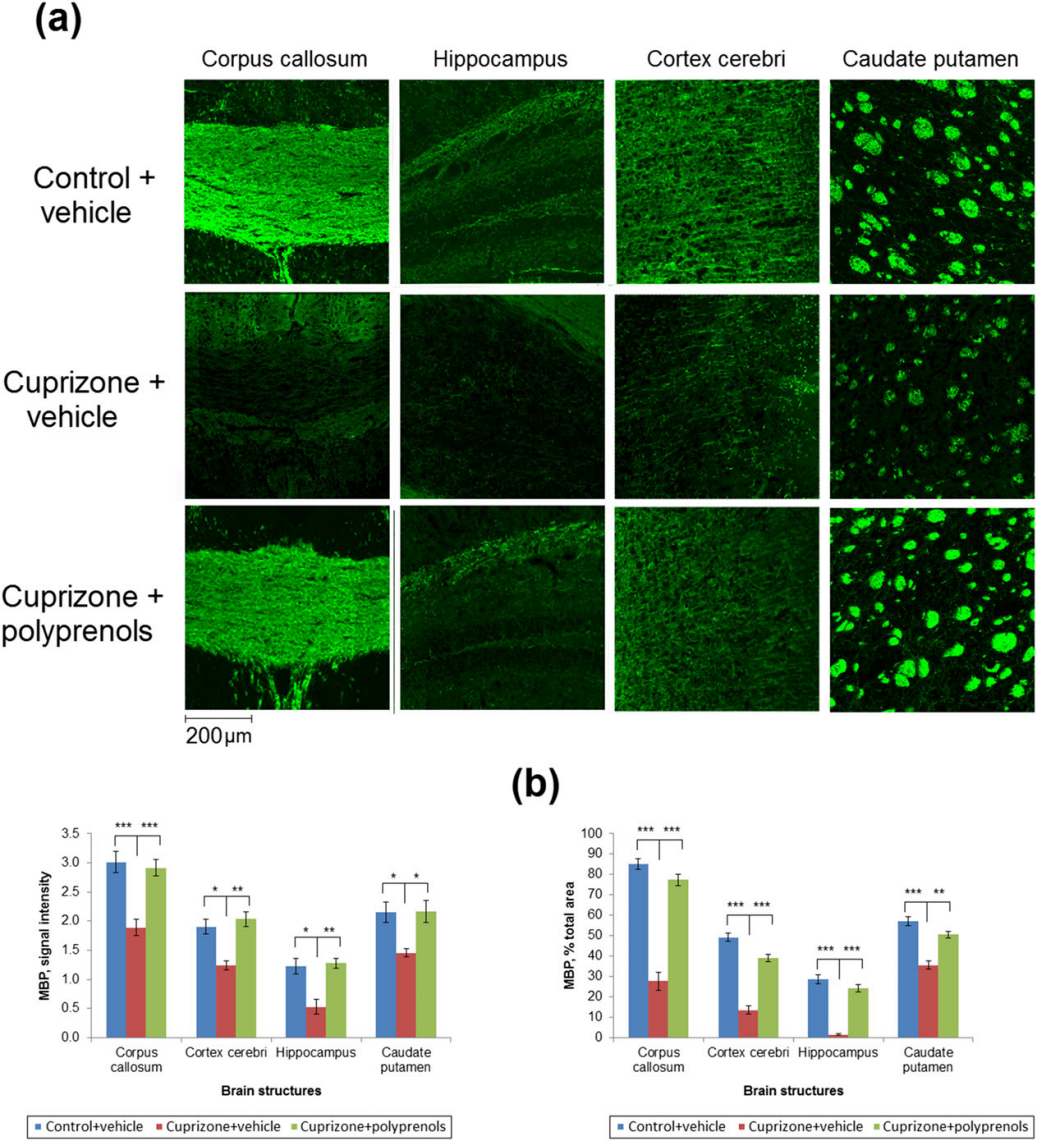
Quantification of myelin in the brains of mice treated and untreated with cuprizone and polyprenols. (a) Representative magnified views of brain sections stained for myelin basic protein (MBP) in mice not treated with cuprizone and injected with vegetable oil vehicle (control + vehicle), cuprizone treated and injected with vegetable oil vehicle (cuprizone + vehicle), and cuprizone treated and injected with polyprenols (cuprizone + polyprenols). (b) Mean MBP signal intensity (left) and percentage of MBP‐positive total area (right) within the investigated structures. Significant differences between the groups according to analysis of variance after Tukey's correction for multiple comparisons: ***p < 0.001, **p < 0.01, *p < 0.05. Bars in the panel (b) represent standard errors of mean [Colour figure can be viewed at wileyonlinelibrary.com]

Representative brain sections stained for MBP from the control + vehicle, cuprizone + vehicle, and cuprizone + polyprenols groups of mice are shown in Figure 2a. Microphotographs of sections from mice from the cuprizone + vehicle group had lower fluorescent signal intensity and tissue contrast in the corpus callosum, cortex, hippocampus, and caudate putamen compared with both the control + vehicle and cuprizone + polyprenols groups (Figure 2a). Quantitative comparison of average MBP signal intensities and total percentage of MBP‐positive area in the corpus callosum, cortex, hippocampus, and caudate putamen between the groups is presented in Figure 2b. Myelin content in the corpus callosum of mice from the cuprizone + vehicle group significantly decreased compared with the control + vehicle group. In contrast, sections from mice treated with cuprizone and polyprenols did not show a significant reduction in the content of MBP in the investigated structures but demonstrated a significant increase in MBP compared with the cuprizone + vehicle group.

### 3.3 Polyprenols decrease oligodendrocyte overproduction

**Figure 3:**
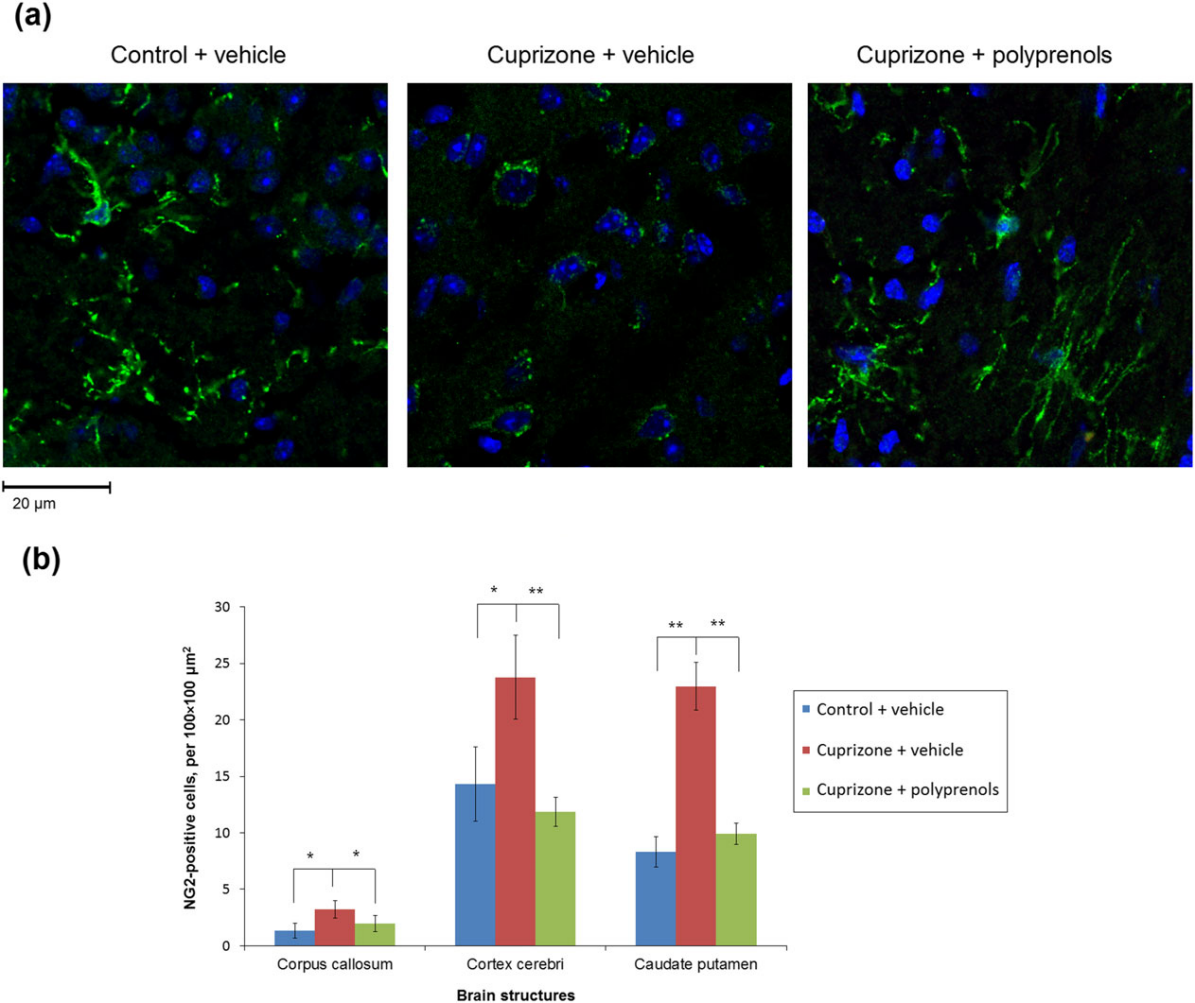
The effect of polyprenols on oligodendrogenesis in cuprizone‐treated mice. (a) Representative microphotographs of neuro‐glial antigen‐2 (NG2)‐stained (green stain) sections in the cortex of the control + vehicle‐, cuprizone + vehicle‐, and cuprizone + polyprenols‐treated mice. (b) Mean NG2‐positive cell count in a series of brain structures of the treatment groups. Significant differences between the groups according to analysis of variance after Tukey's correction for multiple comparisons: **p < 0.01, *p < 0.05. Bars in the panel (b) represent standard errors of mean [Colour figure can be viewed at wileyonlinelibrary.com]

The effect of cuprizone and polyprenol treatment on oligodendrogenesis in the mouse brain was examined using staining against NG2 (Figure 3). Representative brain sections stained for NG2 from the control + vehicle, cuprizone + vehicle, and cuprizone + polyprenols groups of mice are displayed in Figure 3a. Despite a large amount of OPCs, the morphology of immature oligodendrocytes in the cuprizone + vehicle group was less developed than OPCs in the control group and the cuprizone + polyprenols group. The immature oligodendrocytes in the former group had shorter, simpler, and less ramified processes. The cuprizone + vehicle group (green staining) showed significantly increased amount of OPCs in the corpus callosum, cortex, and caudate putamen compared with both the cuprizone + polyprenols and control + vehicle groups (Figure 3b). The number of NG2‐positive cells in the cuprizone + polyprenols group was not significantly different from the control + vehicle group.

### 3.4 Polyprenols restore impaired neurogenesis

**Figure 4:**
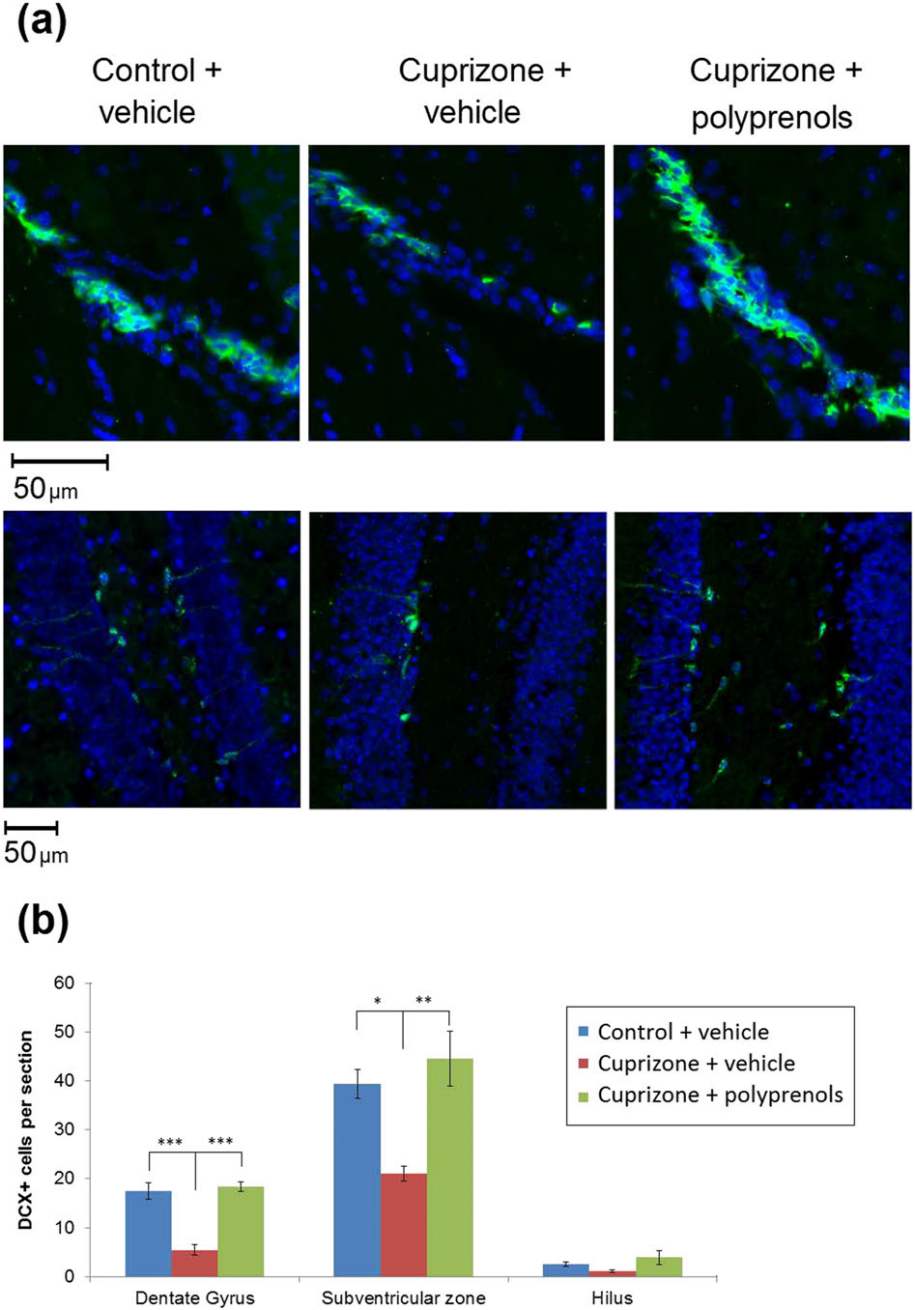
The effect of polyprenols on neurogenesis in cuprizone‐treated mice. (a) Representative microphotographs of doublecortin (DCX)‐stained (green staining) sections in the SVZ and the dentate gyrus of the hippocampus. (b) Mean DCX‐positive cell count in neurogenic zones of the treatment groups. Significant differences between the groups according to analysis of variance after Tukey's correction for multiple comparisons: ***p < 0.001, **p < 0.01, *p < 0.05. Bars in the panel (b) represent standard errors of mean [Colour figure can be viewed at wileyonlinelibrary.com]

The effect of cuprizone and polyprenol treatment on neurogenesis in the mouse brain was examined using immunostaining against DCX (Figure 4). Representative brain sections stained for DCX (green staining) in zones of active neurogenesis in the control + vehicle, cuprizone + vehicle, and cuprizone + polyprenols groups of mice are shown in Figure 4a. Cuprizone treatment significantly reduced the number of DCX‐positive immature neurons in both active zones of neurogenesis, the SVZ and subgranular zone (Figure 4b). In the cuprizone + polyprenols group, neurogenesis did not differ significantly from that in the control group.

## 4 DISCUSSION

This study shows that damage to the mouse brain tissues caused by 10 weeks of cuprizone administration including demyelination, overproduction of immature oligodendrocytes, and impaired neurogenesis can be substantially reduced with polyprenol treatment from Weeks 6 to 10. Particularly, the MBP content in all investigated brain structures of animals treated with polyprenols was significantly greater than that observed in the cuprizone‐treated mice with sham injections and did not differ from the control group. Polyprenol injections also returned the amounts of OPCs and immature neurons to the normal levels. The findings of immunohistochemical studies are corroborated by the results of the open‐field tests, which demonstrated that the behavioral deficit caused by cuprizone intoxication was greatly alleviated under the influence of polyprenols. Specifically, polyprenol treatment completely reversed anxiety‐like behavior and improved locomotor activity, though it did not affect vertical activity and grooming.

The mechanism of halting the neurotoxic effect of cuprizone by polyprenols may be related to restoring impaired oligodendrogenesis and neurogenesis and promoting remyelination. Alternatively, polyprenols may prevent the development of the chronic demyelination state and neurodegeneration, being administered concurrently with cuprizone. While the elucidation of the exact mechanism of action of polyprenols was beyond the scope of this pilot study, our results unambiguously demonstrate their beneficial effect in the cuprizone model of MS. More research is necessary to identify whether these therapeutic benefits are either related to their ability to promote remyelination and stimulate restorative processes in the CNS or reflect their general neuroprotective effect.

Our observations in the cuprizone‐treated animals without polyprenol administration are in line with the literature and confirm successful induction of the demyelination model. The myelin content in the corpus callosum, cortex, hippocampus, and caudate putamen, according to the quantitative MBP immunofluorescence analysis, was significantly decreased in this group, which is in agreement with the previous studies (Gudi et al., 2014; Khodanovich et al., 2016; Khodanovich et al., 2017; Kipp et al., 2009; Koutsoudaki et al., 2009; Pott et al., 2009). The behavioral deficit observed in the cuprizone‐treated group supports the presence of toxic demyelination. Cuprizone altered the open‐field activity towards decreasing locomotion and anxiety‐like behavior. Similar changes in the behavior of cuprizone‐treated mice were found in the previous studies (Franco‐Pons, Torrente, Colomina, & Vilella, 2007). Furthermore, the recent findings of impaired maturation of oligodendrocytes (Xing et al., 2014; Q. Yu et al., 2017) and neurogenesis (Abe et al., 2015; Hillis, Davies, Mundim, Al‐Dalahmah, & Szele, 2016) caused by cuprizone intoxication were reproduced in our model. It is known that cuprizone‐induced demyelination causes oligodendrocyte overproduction (Q. Yu et al., 2017) but maturation is delayed (Xing et al., 2014). The increased subpopulation of OPCs mostly originates from the SVZ and migrates to demyelinated areas (Xing et al., 2014). We observed an increased amount of OPCs as indicated by the NG2 marker in demyelinated brain structures in agreement with the literature (Xing et al., 2014, Q. Yu et al., 2017). Similar to the recent studies (Abe et al., 2015; Hillis et al., 2016), we have also found that cuprizone impairs neurogenesis in both neurogenic niches, SVZ and subgranular layer of the dentate gyrus, as indicated by the diminished number of DCX‐positive young neurons.

Several earlier clinical and preclinical studies have demonstrated beneficial effects of polyprenols in certain neuropsychiatric conditions including chronic alcoholism (Soultanov et al., 2010; Wang et al., 2014), cognitive impairment and depression in the models of Alzheimer's disease (Fedotova et al., 2012; Fedotova et al., 2016; Soultanov et al., 2017), and d‐galactose‐induced accelerated aging model (Surmacz & Swiezewska, 2011). Although little is known about mechanisms of brain‐specific biological activity of polyprenols, one may speculate that some of their properties confirmed earlier could be responsible for the positive therapeutic effect in this and previous studies. Polyprenols can accumulate at relatively high levels within eukaryotic organisms (Hartley & Imperiali, 2012). Unsaturated plant polyprenols and dolichols are known as potent antioxidants (Cavallini et al., 2016; Swiezewska & Danikiewicz, 2005). Either directly or after conversion to dolichols in eukaryotic cells via polyprenol reductase pathway (Swiezewska & Danikiewicz, 2005), polyprenols may work as scavengers for reactive oxygen and nitrogen species, thus protecting cells from the oxidative stress. Mitochondrial dysfunction caused by oxidative injury is considered a primary mechanism triggering oligodendrocyte apoptosis in cuprizone intoxication (Praet et al., 2014). Notably, oligodendrocytes are known as the most vulnerable to oxidative damage neural cell population (Praet et al., 2014). Oxidative mitochondrial injury associated with microglial activation is also an important mechanism responsible for chronic demyelination, axonal damage, and neurodegeneration in MS as well as other neurodegenerative diseases (Witte, Geurts, de Vries, van der Valk, & van Horssen, 2010). For this reason, various antioxidant therapies in MS are actively investigated (Spain et al., 2017). Another possible explanation of the effect of polyprenols on myelination can be related to their capability of bioconversion to dolichols (Swiezewska & Danikiewicz, 2005), which are known as critical precursors in the biosynthesis of myelin glycoproteins (Uyemura, Horie, Suzuki, & Kitamura, 1981; Volpe, Sakakihara, & Ishii, 1987). As such, remyelination may cause an increased consumption of dolichol derivatives, similar to myelination during the brain development (Volpe et al., 1987), and their exogenous supplementation may be beneficial for myelin recovery.

This study has several limitations. First, the effects of cuprizone intoxication and polyprenol treatment were evaluated at a single time point. More time points will be necessary in future studies to separately assess the action of polyprenols during acute and chronic demyelination stages. Second, the study did not involve a control group with polyprenol treatment only. However, it is extremely unlikely that inclusion of the additional control group could change the conclusions of this study, because toxicity of polyprenols is known to be negligible (Wang et al., 2014). Third, we used the outbred CD‐1 mice, which are common for toxicology and pharmacology research (Festing, 2014) but more resistant to cuprizone demyelination as compared with the typically used for this model inbred C57BL/6 strain (Q. Yu et al., 2017). Although both demyelination and the polyprenol treatment effect were successfully demonstrated in this study, our results may need to be replicated in a better characterized C57BL/6 murine model. Finally, a more extensive panel of markers and additional gene expression studies would be needed to identify mechanisms of the therapeutic effect of polyprenols, including, in particular, evaluation of apoptosis, glial proliferation, inflammation, and immune responses. These aspects were beyond the scope of this exploratory study and need to be addressed in future works.

## 5 CONCLUSIONS

In summary, this study demonstrated that plant polyprenols have a potential to repair cuprizone‐induced damage to the mouse brain. Our results suggest that polyprenols can halt demyelination, recover suppressed neurogenesis, and mitigate reactive overproduction of immature oligodendrocytes caused by cuprizone neurotoxicity. Given the lack of effective remyelination therapies for MS, polyprenols should be fast‐tracked for further testing in other animal models and humans.

## CONFLICT OF INTEREST

Dr. Trusov is an employee of Prenolica Limited (formerly Solagran Limited), the manufacturer of the polyprenol preparation (Ropren®) used in this study. The remaining authors declare no conflict of interest.

## References

[ptr6327-bib-0001] Abe, H. , Tanaka, T. , Kimura, M. , Mizukami, S. , Saito, F. , Imatanaka, N. , … Shibutani, M. (2015). Cuprizone decreases intermediate and late‐stage progenitor cells in hippocampal neurogenesis of rats in a framework of 28‐day oral dose toxicity study. Toxicology and Applied Pharmacology, 287, 210–221. 10.1016/J.TAAP.2015.06.005 26057786

[ptr6327-bib-0002] Acs, P. , Kipp, M. , Norkute, A. , Johann, S. , Clarner, T. , Braun, A. , … Beyer, C. (2009). 17β‐estradiol and progesterone prevent cuprizone provoked demyelination of corpus callosum in male mice. Glia, 57(8), 807–814. 10.1002/glia.20806 19031445

[ptr6327-bib-0003] Bakker, D. , & Ludwin, S. (1987). Blood‐brain barrier permeability during cuprizone‐induced demyelination. Implications for the pathogenesis of immune‐mediated demyelinating diseases. Journal of the Neurological Sciences, 78(2), 125–137. 3553434 10.1016/0022-510x(87)90055-4

[ptr6327-bib-0004] Barnett, M. , & Prineas, J. (2004). Relapsing and remitting multiple sclerosis: Pathology of the newly forming lesion. Annals of Neurology, 55(4), 458–468. 10.1002/ana.20016 15048884

[ptr6327-bib-0005] Cavallini, G. , Sgarbossa, A. , Parentini, I. , Bizzarri, R. , Donati, A. , Lenci, F. , & Bergamini, E. (2016). Dolichol: A component of the cellular antioxidant machinery. Lipids, 51(4), 477–486. 10.1007/s11745-016-4137-x. Epub 2016 Mar 11 26968401

[ptr6327-bib-0006] Chojnacki, T. , & Dallner, G. (1988). The biological role of dolichol. The Biochemical Journal, 251, 1–9. 10.1042/bj2510001 3291859 PMC1148956

[ptr6327-bib-0007] Deshmukh, V. , Tardif, V. , Lyssiotis, C. , Green, C. , Kerman, B. , Kim, H. , … Lairson, L. (2013). A regenerative approach to the treatment of multiple sclerosis. Nature, 502(7471), 327–332. 10.1038/nature12647 24107995 PMC4431622

[ptr6327-bib-0008] Ercan, E. , Han, J. , Nardo, A. , Winden, K. , Han, M.‐J. , Hoyo, L. , … Sahin, M. (2017). Neuronal CTGF/CCN2 negatively regulates myelination in a mouse model of tuberous sclerosis complex. Journal of Experimental Medicine, 6, 214(3), 681–697. 10.1084/jem.20160446. Epub Feb 9 28183733 PMC5339668

[ptr6327-bib-0009] Fedotova, J. , Soultanov, V. , Nikitina, T. , Roschin, V. , & Ordayn, N. (2012). Ropren® is a polyprenol preparation from coniferous plants that ameliorates cognitive deficiency in a rat model of beta‐amyloid peptide‐(25–35)‐induced amnesia. Phytomedicine, 19, 451–456. 10.1016/J.PHYMED.2011.09.073 22305275

[ptr6327-bib-0010] Fedotova, J. , Soultanov, V. , Nikitina, T. , Roschin, V. , Ordyan, N. , & Hritcu, L. (2016). Cognitive‐enhancing activities of the polyprenol preparation Ropren® in gonadectomized β‐amyloid (25–35) rat model of Alzheimer's disease. Physiology & Behavior, 157, 55–62. 10.1016/J.PHYSBEH.2016.01.035 26821186

[ptr6327-bib-0011] Festing, M. (2014). Evidence should trump intuition by preferring inbred strains to outbred stocks in preclinical research. National Research Council, Institute of Laboratory Animal Resources Journal, 55, 399–404. 10.1093/ilar/ilu036 25541542

[ptr6327-bib-0012] Franco‐Pons, N. , Torrente, M. , Colomina, M. , & Vilella, E. (2007). Behavioral deficits in the cuprizone‐induced murine model of demyelination/remyelination. Toxicology Letters, 169(3), 205–213. 10.1016/j.toxlet.2007.01.010 17317045

[ptr6327-bib-0013] Gudi, V. , Gingele, S. , Skripuletz, T. , & Stange, M. (2014). Glial response during cuprizone‐induced de‐ and remyelination in the CNS: Lessons learned. Frontiers in Cellular Neuroscience, 8, 73. 24659953 10.3389/fncel.2014.00073 PMC3952085

[ptr6327-bib-0014] Hartley, M. D. , & Imperiali, B. (2012). At the membrane frontier: A prospectus on the remarkable evolutionary conservation of polyprenols and polyprenyl‐phosphates. Archives of Biochemistry and Biophysics, 517, 83–97. 10.1016/J.ABB.2011.10.018 22093697 PMC3253937

[ptr6327-bib-0015] Hillis, J. , Davies, J. , Mundim, M. , Al‐Dalahmah, O. , & Szele, F. (2016). Cuprizone demyelination induces a unique inflammatory response in the subventricular zone. Journal of Neuroinflammation, 13, 1–15. 10.1186/s12974-016-0651-2 27550173 PMC4994223

[ptr6327-bib-0016] Iwasa, K. , Yamamoto, S. , Takahashi, M. , Suzuki, S. , Yagishita, S. , Awaji, T. , … Yoshikawa, K. (2014). Prostaglandin F2α FP receptor inhibitor reduces demyelination and motor dysfunction in a cuprizone‐induced multiple sclerosis mouse model. Prostaglandins, Leukotrienes, and Essential Fatty Acids, 91(5), 175–182. 10.1016/j.plefa.2014.08.004. Epub 2014 Sep 6 25224839

[ptr6327-bib-0017] Jakobsson, A. , Swiezewska, E. , Chojnacki, T. , & Dallner, G. (1989). Uptake and modification of dietary polyprenols and dolichols in rat liver. Federation of European Biochemical Societies Letters, 255, 32–36. 10.1016/0014-5793(89)81055-5 2507352

[ptr6327-bib-0018] Khodanovich, M. , Glazacheva, V. , Pan, E. , Akulov, A. , Krutenkova, E. , Trusov, V. , & Yarnykh, V. (2016). MRI study of the cuprizone‐induced mouse model of multiple sclerosis: Demyelination is not found after co‐treatment with polyprenols (long‐chain isoprenoid alcohols). Journal of Physics: Conference Series, 677, 8–14. 10.1088/1742-6596/677/1/012007

[ptr6327-bib-0019] Khodanovich, M. , Sorokina, I. , Glazacheva, V. , Akulov, A. , Nemirovich‐Danchenko, N. , Romashchenko, A. , … Yarnykh, V. (2017). Histological validation of fast macromolecular proton fraction mapping as a quantitative myelin imaging method in the cuprizone demyelination model. Scientific Reports, 7, 1–12. 10.1038/srep46686 28436460 PMC5402392

[ptr6327-bib-0020] Kipp, M. , Clarner, T. , Dang, J. , Copray, S. , & Beyer, C. (2009). The cuprizone animal model: New insights into an old story. Acta Neuropathologica, 118, 723–736. 10.1007/s00401-009-0591-3 19763593

[ptr6327-bib-0021] Koutsoudaki, P. N. , Skripuletz, T. , Gudi, V. , Moharregh‐Khiabani, D. , Hildebrandt, H. , Trebst, C. , & Stangel, M. (2009). Demyelination of the hippocampus is prominent in the cuprizone model. Neuroscience Letters, 451, 83–88. 10.1016/J.NEULET.2008.11.058 19084049

[ptr6327-bib-0022] Lucchinetti, C. , Brück, W. , Parisi, J. , Scheithauer, B. , Rodriguez, M. , & Lassmann, H. (2000). Heterogeneity of multiple sclerosis lesions: Implications for the pathogenesis of demyelination. Annals of Neurology, 47, 707–717. 10.1002/1531-8249(200006)47:6<707::AID-ANA3>3.0.CO;2-Q 10852536

[ptr6327-bib-0023] Mojaverrostami, S. , Bojnordi, M. , Ghasemi‐Kasman, M. , Ebrahimzadeh, M. , & Hamidabadi, H. (2018). Review of herbal therapy in multiple sclerosis. Advanced Pharmaceutical Bulletin, 8, 4 10.15171/apb.2018.066–590. PMC6311642 30607330

[ptr6327-bib-0024] Paxinos, G. , & Franklin, K. (2001). The mouse brain in stereotaxic coordinates (4th ed.). San Diego: Academic Press.

[ptr6327-bib-0025] Pott, F. , Gingele, S. , Clarner, T. , Dang, J. , Baumgartner, W. , Beyer, C. , & Kipp, M. (2009). Cuprizone effect on myelination, astrogliosis and microglia attraction in the mouse basal ganglia. Brain Research, 1305, 137–149. 10.1016/J.BRAINRES.2009.09.084 19799876

[ptr6327-bib-0026] Praet, J. , Guglielmetti, C. , Berneman, Z. , Van der Linden, A. , & Ponsaerts, P. (2014). Cellular and molecular neuropathology of the cuprizone mouse model: Clinical relevance for multiple sclerosis. Neuroscience & Biobehavioral Reviews, 47, 485–505. 10.1016/j.neubiorev.2014.10.004 25445182

[ptr6327-bib-0027] Pronin, A. , Grigorieva, E. , Sanin, A. , Narovlyansky, A. , Ozherelkov, S. , Deyeva, A. , … Najid, A. (2002). Polyprenols as possible factors that determine an instructive role of the innate immunity in the acquired immune response. Russian Journal of Immunolojy, 7, 135–142. 12687256

[ptr6327-bib-0028] Roslinska, M. , Walinska, K. , Swiezewska, E. , & Chojnacki, T. (2002). Plant long‐chain polyprenols as chemotaxonomic markers. Dendrbiology, 47, 41–50. 11544679

[ptr6327-bib-0030] Soultanov, V. , Agishev, V. , Monakhova, I. , Mokhovikova, I. , Kulikov, A. , Roschin, V. , & Nikitina, T. (2010). Ropren ® improves liver and pancreatic function in patients with chronic alcoholism. Gastroenterologia Sankt‐Peterburga, 4, 12–17.

[ptr6327-bib-0031] Soultanov, V. , Fedotova, J. , Nikitina, T. , Roschin, V. , Ordyan, N. , & Hritcu, L. (2017). Antidepressant‐like effect of Ropren® in β‐amyloid‐(25–35) rat model of Alzheimer's disease with altered levels of androgens. Molecular Neurobiology, 54, 2611–2621. 10.1007/s12035-016-9848-8. Epub 2016 Mar 1912 26993300

[ptr6327-bib-0032] Spain, R. , Powers, K. , Murchison, C. , Heriza, E. , Winges, K. , Yadav, V. , … Bourdette, D. (2017). Lipoic acid in secondary progressive MS: A randomized controlled pilot trial. Neurol Neuroimmunol Neuroinflamm, 28, 4(5), e374 10.1212/NXI.000000000000037 28680916 PMC5489387

[ptr6327-bib-0033] Surmacz, L. , & Swiezewska, E. (2011). Polyisoprenoids—Secondary metabolites or physiologically important superlipids? Biochemical and Biophysical Research Communications, 407(4), 627–632. 10.1016/j.bbrc.2011.03.059 21419101

[ptr6327-bib-0034] Swiezewska, E. , & Danikiewicz, W. (2005). Polyisoprenoids: Structure, biosynthesis and function. Progress in Lipid Research, 44(4), 235–258. 10.1016/j.plipres.2005.05.002 16019076

[ptr6327-bib-0035] Uyemura, K. , Horie, K. , Suzuki, M. , & Kitamura, K. (1981). Glycosylation of myelin glycoproteins in peripheral nerve via lipid intermediates. Neurochemical Research, 6(9), 959–968. 10.1007/BF00965027 7322261

[ptr6327-bib-0036] Vega‐Riquer, J. , Mendez‐Victoriano, G. , Morales‐Luckie, R. , & Gonzalez‐Perez, O. (2017). Five decades of cuprizone, an updated model to replicate demyelinating diseases. Current Neuropharmacology, 17, 10.2174/1570159X15666170717120343. [Epub ahead of print], 129–141. PMC6343207 28714395

[ptr6327-bib-0037] Veto, S. , Acs, P. , Bauer, J. , Lassmann, H. , Berente, Z. , Setalo, G. , … Illes, Z. (2010). Inhibiting poly (ADP‐ribose) polymerase: A potential therapy against oligodendrocyte death. Brain, 133(3), 822–834. 10.1093/brain/awp337 20157013 PMC2964508

[ptr6327-bib-0038] Volpe, J. , Sakakihara, Y. , & Ishii, S. (1987). Dolichol‐linked glycoprotein synthesis in developing mammalian brain: Maturational changes of the N‐acetylglucosaminylphosphotransferase. Brain Research, 430(2), 277–284. 10.1016/0165-3806(87)90160-X 3038274

[ptr6327-bib-0039] Walinska, K. (2004). Comparison of the influence of the polyprenol structure on model membranes. Desalination, 163, 239–245. 10.1016/S0011-9164(04)90195-6

[ptr6327-bib-0040] Wang, C. , He, L. , Yan, M. , Zheng, G. Y. , & Liu, X. Y. (2014). Effects of polyprenols from pine needles of *Pinus massoniana* on ameliorating cognitive impairment in a d‐galactose‐induced mouse model. Age (Omaha), 36, 36 10.1007/s11357-014-9676-6 PMC4150899 24981114

[ptr6327-bib-0041] Wang, C. , Yuan, J. , Li, W. , Zhang, H. , & Ye, J. (2015). In vivo and in vitro toxicity evaluation of polyprenols extracted from Ginkgo biloba L. leaves. Molecules, 11(20), 22257–22271. 10.3390/molecules201219839 PMC6332170 26690406

[ptr6327-bib-0042] Witte, M. , Geurts, J. , de Vries, H. , van der Valk, P. , & van Horssen, J. (2010). Mitochondrial dysfunction: A potential link between neuroinflammation and neurodegeneration? Mitochondrion, 10(5), 411–418. 10.1016/j.mito.2010.05.014 20573557

[ptr6327-bib-0043] Xing, Y. L. , Roth, P. T. , Stratton, J. A. S. , Chuang, B. H. A. , Danne, J. , Ellis, S. L. , … Merson, T. D. (2014). Adult neural precursor cells from the subventricular zone contribute significantly to oligodendrocyte regeneration and remyelination. Journal of Neuroscience Research, 34, 14128–14146. 10.1523/JNEUROSCI.3491-13.2014 PMC6705285 25319708

[ptr6327-bib-0044] Yang, L. , Wang, C. , Ye, J. , & Li, H. (2011). Hepatoprotective effects of polyprenols from Ginkgo biloba L. leaves on CCl4‐induced hepatotoxicity in rats. Fitoterapia, 82, 834–840. 10.1016/J.FITOTE.2011.04.009 21596107

[ptr6327-bib-0045] Yu, J. , Wang, Y. , Qian, H. , Zhao, Y. , Liu, B. , Fu, C. , et al. (2012). Polyprenols from Taxus chinensis var. mairei prevent the development of CCl4‐induced liver fibrosis in rats. Journal of Ethnopharmacology, 142, 151–160. 10.1016/j.jep.2012.04.030 22543175

[ptr6327-bib-0046] Yu, Q. , Hui, R. , Park, J. , Huang, Y. , Kusnecov, A. , Dreyfus, C. , & Zhou, R. (2017). Strain differences in cuprizone induced demyelination. Cell & Bioscience, 7, 1–11. 10.1186/s13578-017-0181-3 29142736 PMC5670722

[ptr6327-bib-0047] Zarshenas, M. , Ansari, R. , Dadbakhsh, A. , & Mohammadi, M. (2018). Review of herbal remedies for multiple sclerosis‐like disorders in traditional Persian medicine (TPM). Current Drug Metabolism, 19, 392–407. 10.2174/1389200219666180305152057 29512456

[ptr6327-bib-0048] Zhang, Q. , Huang, L. , Zhang, C. , Xie, P. , Zhang, Y. , Ding, S. , & Xu, F. (2015). Synthesis and biological activity of polyprenols. Fitoterapia, 106, 184–193. 10.1016/J.FITOTE.2015.09.008 26358482

[ptr6327-bib-0029] Zhang, Y. , Xu, H. , Jiang, W. , Xiao, L. , Yan, B. , He, J. , … Li, X. M. (2018). Quetiapine alleviates the cuprizone‐induced white matter pathology in the brain of C57BL/6 mouse. Schizophrenia Research, 106(2–3), 182–191. 10.1016/j.schres.2008.09.013 18938062

